# Evidence supporting dissimilatory and assimilatory lignin degradation in *Enterobacter lignolyticus* SCF1

**DOI:** 10.3389/fmicb.2013.00280

**Published:** 2013-09-19

**Authors:** Kristen M. DeAngelis, Deepak Sharma, Rebecca Varney, Blake Simmons, Nancy G. Isern, Lye Meng Markilllie, Carrie Nicora, Angela D. Norbeck, Ronald C. Taylor, Joshua T. Aldrich, Errol W. Robinson

**Affiliations:** ^1^Department of Microbiology, University of Massachusetts AmherstAmherst, MA, USA; ^2^Deconstruction Division, Joint BioEnergy InstituteEmeryville, CA, USA; ^3^Sandia National LaboratoryLivermore, CA, USA; ^4^Envrionmental Molecular Sciences LaboratoryRichland, WA, USA

**Keywords:** decomposition, anaerobic metabolism, phenol degradation, 4-hydroxyphenylacetate degradation pathway, catalase/peroxidase enzymes, glutathione S-transferase proteins

## Abstract

Lignocellulosic biofuels are promising as sustainable alternative fuels, but lignin inhibits access of enzymes to cellulose, and by-products of lignin degradation can be toxic to cells. The fast growth, high efficiency and specificity of enzymes employed in the anaerobic litter deconstruction carried out by tropical soil bacteria make these organisms useful templates for improving biofuel production. The facultative anaerobe *Enterobacter lignolyticus* SCF1 was initially cultivated from Cloud Forest soils in the Luquillo Experimental Forest in Puerto Rico, based on anaerobic growth on lignin as sole carbon source. The source of the isolate was tropical forest soils that decompose litter rapidly with low and fluctuating redox potentials, where bacteria using oxygen-independent enzymes likely play an important role in decomposition. We have used transcriptomics and proteomics to examine the observed increased growth of SCF1 grown on media amended with lignin compared to unamended growth. Proteomics suggested accelerated xylose uptake and metabolism under lignin-amended growth, with up-regulation of proteins involved in lignin degradation via the 4-hydroxyphenylacetate degradation pathway, catalase/peroxidase enzymes, and the glutathione biosynthesis and glutathione S-transferase (GST) proteins. We also observed increased production of NADH-quinone oxidoreductase, other electron transport chain proteins, and ATP synthase and ATP-binding cassette (ABC) transporters. This suggested the use of lignin as terminal electron acceptor. We detected significant lignin degradation over time by absorbance, and also used metabolomics to demonstrate moderately significant decreased xylose concentrations as well as increased metabolic products acetate and formate in stationary phase in lignin-amended compared to unamended growth conditions. Our data show the advantages of a multi-omics approach toward providing insights as to how lignin may be used in nature by microorganisms coping with poor carbon availability.

## Introduction

Lignocellulose is the most abundant biopolymer on earth, and a recent joint analysis by the DOE and USDA shows that there is sufficient national supply to make lignocellulosic biofuels technically feasible (Perlack, 2005). Development of renewable, sustainable biofuels from plant feedstock material has emerged as a key goal of the US Department of Energy. The use of lignocellulose as a renewable energy source has many advantages, above all that lignocellulose production is domestic and independent of food agriculture (Lee et al., 2008). The deconstruction of plant biomass is a key first step in the conversion of plant sugars to biofuels, though this step has posed a great challenge to making biofuels economically viable. The major hurdles involve lignin occlusion of cellulose, as well as lignin derivatives that inhibit lignocellulose deconstruction and fuel synthesis (Lee et al., 2008). Lignin comprises up to 25% of plant biomass (Wei et al., 2009), and as such is an abundant and potentially valuable waste stream that is currently burned to produce energy as heat (Jaeger and Eggert, 2002). Our primary goal is to improve biofuel production through better saccharification of pretreated feedstock (switchgrass) from pathways and enzymes of anaerobic bacterial lignin degraders. By characterizing anaerobic lignin degradation in the bacterium *Enterobacter lignolyticus* SCF1, we may be able to incorporate these enzymes and pathways into metabolic engineering of biofuel- and biodiesel-producing bacteria. These discoveries also promise to provide insight to the natural processes of bacterial lignin decomposition.

Tropical soils are responsible for near complete decomposition of leaf plant litter in as little as 18 months (Parton et al., 2007). There is an apparent contradiction of tropical forest soils, where rapid and efficient lignocellulose mineralization proceeds rapidly under low or fluctuating redox conditions. Rapid decomposition may be fueled by fluctuating redox conditions that regenerate oxidized iron; up to 10% of tropical bacteria are capable of iron reduction (Dubinsky et al., 2010). Resident microbes are adapted to the low and fluctuating redox potential in the soil (Silver et al., 1999, in press; Pett-Ridge et al., 2006), in contrast to temperate systems where oxidative enzyme activities are rate-limiting for decomposition (Paul and Clark, 1996; Freeman et al., 2001; Fierer et al., 2009). Thus wet tropical soils are attractive targets for discovery of bacterial lignin-degraders, which would be amenable to industrial engineering and efficient for removing lignin inhibitors to cellulose availability for biofuels.

Though fungi are considered primary decomposers, capabilities for genetic manipulation fungi are not as well-developed as for other biological systems, and current fungal enzymes of commercial interest have been too non-specific and too expensive to produce industrially. Fungi have well-characterized mechanisms for breaking open lignin phenol rings via oxygen free-radicals generated by dioxygenase enzymes (Sánchez, 2009; Fujii et al., 2013). Though fungi are thought to dominate decomposition in terrestrial ecosystems, few fungi are known to be able to tolerate the frequent anoxic conditions characteristic of tropical forest soils (Boer et al., 2005; Baldrian and Valášková, 2008). Based on previous observations of considerable anaerobic decomposition in the lab and field (Pett-Ridge and Firestone, 2005; DeAngelis et al., 2010a,b, 2012), we suspect that tropical soil bacteria play a larger role in decomposition under anaerobic and fluctuating redox conditions.

Few bacteria are known to degrade lignin, and even fewer anaerobically. Known potential lignin-degrading bacteria are in the groups α-proteobacteria, γ-proteobacteria, Firmicutes and Actinomycetes (Bugg et al., 2011b) and most bacteria employ extracellular peroxidases, which require oxygen availability (Bugg et al., 2011a). For example, the novel isolates in the phylum Firmicutes *Bacillus pumilus* strain C6 and *Bacillus atrophaeus* strain B7 were identified to have very high laccase activity as well as the ability to aerobically degrade Kraft lignin and the lignin model dimer guaiacylglycerol-b-guaiacyl ether (Huang et al., 2013). Many bacterial processes have been successfully engineered into consolidated bioprocessing for biofuels, such as cellulose conversion to sugars (saccharification) and ionic liquid pretreatment tolerance (Blanch et al., 2008; Lee et al., 2008; Singh et al., 2009), with an emerging role for bacterial lignin degradation (Bugg et al., 2011b). Among anaerobic bacterial lignin or phenol degraders, *Sphingomonas paucimobilis* SYK-6 produces a β-aryl etherase (Masai et al., 2007), and *Rhodococcus* sp. RHA1 contains a β-ketoadipate pathway (McLeod et al., 2006); *Kocuria* and *Staphylococcus* also likely degrade phenol (DeRito et al., 2005). Another Enterobacter species, *E. solis* strain LF7, was isolated from tropical forest soils in Peru based on its ability to degrade alkali lignin as a sole C source under aerobic growth conditions (Manter et al., 2011). *E. solis* strain LF7 and our strain *E. lignolyticus* SCF1 share 97% sequence identify for their 16S ribosomal RNA genes, which is a relatively low homology for the Enterobacteraceae. *E. lignolyticus* SCF1 is a γ-proteobacteria, and a novel isolate in the class Enterobacterales which has been previously shown to be capable of anaerobic lignin-degradation (DeAngelis et al., 2011), though the mechanisms are unknown.

The facultative anaerobe *E. lignolyticus* (formerly *cloaceae)* SCF1 was originally isolated on lignin as sole C source from soil in the El Yunque Experimental Forest, Puerto Rico, USA (DeAngelis et al., 2011). The genome sequence of SCF1 suggested that two multi-copper oxidases (putative laccases) and a putative peroxidase may be involved in lignin degradation, with one or more glutathione S-transferase (GST) proteins involved in cleaving β-aryl ether linkages. This is the case with LigE/LigF in *S. paucimobilis*, where lignin is degraded by way of the protocatechuate pathway, catalyzed in part by the protocatechuate 4,5-dioxygenase enzyme LigB and the extradiol dioxygenase LigZ (Masai et al., 2007; Peng et al., 2008). However, SCF1 does not posses the core protocatechuate and 3-O-methylgallate degradation pathways found in *S. paucimobilis*. Instead, lignin catabolism seemed likely to proceed via homoprotocatechuate through the 4-hydroxyphenylacetate degradation pathway, a gene cluster that is conserved among the *Enterobacter* and *Klebsiella* (Bugg et al., 2011a). In this study, we use proteomics, transcriptomics, metabolomics analysis and measures of enzyme activities to characterize the mechanism by which *E. lignolyticus* SCF1 is able to degrade lignin during anaerobic growth conditions.

## Methods

### Cultivation conditions

For the lignin degradation experiment, cultures were initially streaked onto 10% tryptic soy broth (TSB), 1.5% agar plates, then transferred after 24 h to 10 ml modified LS4D minimal media (also referred to as xylose minimal media), which consists of 8 mM MgCl_2_, 20 mM NH_4_Cl, 2.2 mM KH_2_PO_4_, 2 mM Tris-Cl, 0.6 mM CaCl-2H_2_0, and 0.8% xylose, buffered to pH 7. These liquid cultures were incubated anaerobically for 24 h, until the optical density at 600 nm achieved about 0.140 OD. At this point, 0.6 ml of cell culture was transferred to 100 ml of xylose minimal media with and without 0.05% lignin. The lignin used in these studies was alkali lignin (Sigma 45-471003), selected based on relative solubility in water and low molecular weight. Cultures were grown anaerobically in serum bottles with 5% hydrogen, 5% CO_2_, and 90% (balance) N_2_ as headspace at 30°C. During the 48 h growth, cell counts (by DAPI direct counts and optical density at 600 nm) and lignin degradation (by change in absorbance at 310 nm) were measured. Samples were immediately placed at −80°C until further analysis. For analyzing supernatants, samples were filtered through a 0.22 um syringe filter into a sterile microplate, with 200 uL of sample in each well covered with sterile, pierce-able foil.

### Oxidative enzyme assays

To perform measurements of oxidative enzyme activity, cells were grown as above in xylose minimal media, and then amended with L-3,4-dihydroxyphenylalanine (L-DOPA). L-DOPA is a lignin analog, where reduction causes a color change detectable colorimetrically (Saiya-Cork et al., 2002). For aerobic analysis, SCF-1 was grown in xylose minimal media broth for 12 h at 30°C with shaking at 200 RPM (for aerobic growth; no shaking for anaerobic growth) until an average OD at 600 nm of 0.9 was reached, indicating late log phase based upon previous growth curves of this organism grown aerobically. For anaerobic analysis, SCF-1 was grown anoxically in xylose minimal media broth for 24 h until an average OD at 600 nm of 0.1 was reached, indicating late log phase based upon previous growth curves of this organism grown anoxically. For phenol oxidase and peroxidase assays, 25 mM L-DOPA substrate was prepared the same day as analysis, with 3% H_2_O_2_ added for peroxidase assays. Phenol oxidase and peroxidase were also measured using 2,2′-azino-bis(3-ethylbenzothiazoline-6-sulphonic acid) (ABTS) based on a published protocol (Floch et al., 2007). The ABTS assays were prepared in the same way as for the L-DOPA assays, where 2 mM ABTS was prepared, and these assays performed only on aerobically grown cells. To measure enzyme activity, 500 uL of cell culture was combined with 500 uL of substrate. Time was recorded from the time substrate was added to cell culture. Measurements were made at absorbance at 460 nm. Each plate contained three biological replicates for each assay, with eight technical replicates (wells) for each. For each assay, negative controls included media, cell culture, and media and substrate, and signal OD was calculated as: [(Assay Value – Blank) – (Reference Standard – Blank)] where the blank was media only, and the reference standard was media + DOPA or ABTS. This accounted for any activity of trace metals in the media (i.e., Mn and Fe). ABTS rates are reported as mU (10^6^ cells)^−1^, which is milliunits of ABTS (or 10^−3^ units) per million cells.

### Proteomics

After 48 h of growth, cells grown in lignin-amended or unamended xylose minimal media (as detailed above) were harvested for proteomics and transcriptomics assays. This time point was chosen based on strong differences observed between lignin degraded and cell growth in amended vs. unamended conditions, with no further growth or significant lignin degradation observed after around this time. For this analysis, three biological replicates of cells grown in lignin-amended and unamended conditions were analyzed. A methanol/chloroform extraction was done on the supernatant to separate the protein, metabolites and lipids. Ice cold (−20°C) cholorform:methanol mix [prepared 2:1 (v/v)] was added to the sample in a 5:1 ratio over sample volume and vigorously vortexed. The sample was then placed on ice for 5 min and then vortexed for 10 s followed by centrifugation at 10,000 xg for 10 min at 4°C. The upper, water soluble metabolite phase and the lower, lipid soluble phase were collected into separate glass vials, and both samples were dried to complete dryness in a speed vac and then stored at −80°C until analysis. The remaining protein interlayer was placed in a fume hood to dry.

The protein pellet was resuspended in 8M urea and assayed with Bicinchoninic acid (BCA) (Thermo Scientific, Rockford, IL) to determine the protein concentration. 10 mM DTT was then added to the sample, sonicated and incubated at 60°C for 30 min with constant shaking at 800 rpm. Samples were then diluted 8-fold for preparation for digestion with 100 mM NH_4_HCO_3_, 1 mM CaCl_2_ and sequencing-grade modified porcine trypsin (Promega, Madison, WI) was added to all protein samples at a 1:50 (w/w) trypsin-to-protein ratio for 3 h at 37°C. The samples were cleaned using Discovery C18 50 mg/1 mL solid phase extraction tubes (Supelco, St.Louis, MO), using the following protocol: 3 mL of methanol was added for conditioning followed by 2 mL of 0.1% TFA in H2O. The samples were then loaded onto each column followed by 4 mL of 95:5: H_2_O:ACN, 0.1% TFA. Samples were eluted with 1 mL 80:20 ACN:H_2_O, 0.1% TFA. The samples were concentrated down to ~30 μL using a Speed Vac and a final was performed to determine the peptide concentration. The samples were then vialed for mass spectrometric analysis.

To generate the AMT database, pooled samples of equal mass from each biological replicate of the lignin and xylose samples were combined and run using a custom built 2D-LC system using two Agilent 1200 nanoflow pumps and one 1200 capillary pump (Agilent Technologies, Santa Clara, CA), various Valco valves (Valco Instruments Co., Houston, TX), and a PAL autosampler (Leap Technologies, Carrboro, NC). Full automation was made possible by custom software that allows for parallel event coordination and therefore near 100% MS duty cycle through use of two trapping columns and two analytical columns. All columns were manufactured in-house by slurry packing media into fused silica (Polymicro Technologies Inc., Phoenix, AZ) using a 1-cm sol-gel frit for media retention [a PNNL variation of Maiolica et al. (2005)]. Samples were run as 15 fractions separated in the 1st dimension by SCX fractionation and reversed-phase separation in the 2nd dimension. Mobile phases consisted of 0.05% ACN in Nano H20 (A) and 500mM Ammonia Formate (B) and 0.1% formic acid in water (A) and 0.1% formic acid in acetonitrile (B) for the 1st and 2nd dimensions respectively. Supplemental Table 1 describes the change in mobile phase for each fraction.

MS analysis was performed using a Velos-LTQ-Orbitrap mass spectrometer (Thermo Scientific, San Jose, CA) outfitted with a custom-built electrospray ionization (ESI) interface. Electrospray emitters were custom made using 150 um o.d. × 20 um i.d. chemically etched fused silica (Kelly et al., 2006). The heated capillary temperature and spray voltage were 300°C and 2.2 kV, respectively. Data was acquired for 100 min, beginning 65 min after sample injection and 15 min into gradient. Orbitrap spectra (AGC 1×106) were collected from 400–2000 m/z at a resolution of 60 k followed by data dependent ion trap CID MS/MS (collision energy 35%, AGC 3×104) of the ten most abundant ions. A dynamic exclusion time of 60 s was used to discriminate against previously analyzed ions.

The quantitative samples were run using a custom HPLC system configured using 65 mL Isco Model 65D syringe pumps (Isco, Inc., Lincoln, NE), 2-position Valco valves (Valco Instruments Co., Houston, TX), and a PAL autosampler (Leap Technologies, Carrboro, NC), allowing for fully automated sample analysis across four separate HPLC columns. Reversed-phase capillary HPLC columns were manufactured in-house by slurry packing 5 μm Jupiter C18 stationary phase (Phenomenex, Torrence, CA) into fused silica (Polymicro Technologies Inc., Phoenix, AZ) using a 0.5 cm sol-gel frit for media retention [a PNNL variation of Maiolica et al. (2005)]. Mobile phases consisted of 0.1% formic acid in water (A) and 0.1% formic acid in acetonitrile (B). The mobile phase flowed through an in-line Degassex DG4400 degasser (Phenomenex, Torrance, CA). The HPLC system was equilibrated at 10k psi with 100% mobile phase A. Fifty min after sample injection the mobile phase was switched to 100% B, which created a near-exponential gradient as mobile phase B displaced A in a 2.5 mL active mixer. A 35 cm length of 360 μm o.d. × 15 μm i.d. fused silica tubing was used to split ~18 μL min^−1^ of flow before it reached the injection valve (5 uL sample loop). The split flow controlled the gradient speed under conditions of constant pressure operation (10 k psi). Flow through the capillary HPLC column when equilibrated to 100% mobile phase A was ~400 nL min^−1^. MS analysis was identical to that of the 2D system.

The Accurate Mass and Time (AMT) tag (Hixson et al., 2006; Monroe et al., 2007) approach was applied to produce quantitative peptide abundance data. This method is an LC-MS approach which matches LC-MS features to a previously generated database using the metrics monoisotopic mass and normalized elution time (NET). Peptide sequences were identified using the SEQUEST v.27 (rev. 12) search engine and then rescored using MS-GF (Mass Spectum-Generating Function) (Kim et al., 2008). The feature database was populated using identifications having an MSGF Score ≤ 1E-9, partially/fully tryptic or protein terminal as well as a peptide prophet probability ≥ 0.5. Features from the 1-D analysis were matched to this database and filtered using a uniqueness probability ≥ 0.51 to ensure specificity of the match.

Peak matching of the 1D data was performed against the AMT database for peptide identification and peptide abundance. Identifications which referenced multiple proteins were removed from the peptide list. The quantitative information was then analyzed using the analysis suite DanteR (Taverner et al., 2012). Within this framework the data were log2 transformed and normalized using median central tendency. Technical replicate abundances were averaged to get the abundance value for each biological replicate and required at least two abundance values to be used. Each protein had its member peptides fit to a linear model treating media and peptide as fixed effects to estimate the effect due to media and *p*-value significance. The generated *p*-values were then adjusted to compensate for multiple comparisons using Benjamini–Hochberg *p*-value correction (Benjamini and Hochberg, 1995). Proteins with a corrected *p*-value ≤ 0.05 were considered significantly differentially regulated. Additionally each peptide was fit to a simple model comparing the effect size and direction due to media and this was compared to that of the protein results to ensure reliability of the protein model.

Metabolic pathway analysis was performed using Pathway Tools software version 16.5 (Karp et al., 2002). Pathway-Genome Database (PGDB) for SCF1 was previously generated (Khudyakov et al., 2012) based on the genome annotation from the Joint Genome Institute's Integrated Microbial Genomics (IMG) system (Markowitz et al., 2010), and supplemented with additional Enzyme Commission numbers from Rapid Annotation using Subsystem Technology (RAST) (Aziz et al., 2008). It has undergone minimal manual curation and may contain some errors, similar to a tier 3 BioCyc PGDB (Karp et al., 2005). Data visualization was performed using omics viewer on Pathway Tool (Paley and Karp, 2006). Proteomics data can be found in the public proteomics repository at omics.pnl.gov via the link http://www.peptideatlas.org/PASS/PASS00294.

### Transcriptomics

Cells were harvested after 48 h growth in lignin-amended or unamended xylose minimal media (as detailed above), in order to analyze transcripts and proteins from the same samples. For this analysis, the same three biological replicates of cells grown in lignin-amended and unamended conditions were analyzed for transcripts as for proteins. RNA was extracted using *Invitrogen* TRIzol® Reagent (cat#15596018), followed by genomic DNA removal and cleaning using Qiagen RNase-Free DNase Set kit (cat#79254) and Qiagen Mini RNeasy™ kit (cat#74104). Agilent 2100 Bioanalyzer was used to assess the integrity of the RNA samples. Only RNA samples having RNA Integrity Number between 8 and 10 were used. For RNA-Sequencing, the Applied Biosystems SOLiDTM Total RNA-Seq kit (catalog number 4445374) was used to generate the cDNA template library. The SOLiDTM EZ Bead system was used to perform emulsion clonal bead amplification to generate bead templates for SOLiDTM platform sequencing. Samples were sequenced on the SOLiDTM 4 platform. The 50-base short read sequences produced by the SOLiDTM 4 sequencer were mapped in color space using SOLiDTM BioScopeTM software version 1.3 using the default settings to map the short reads onto *E. lignolyticus* SCF1 (NC_014618) reference genome; both the fasta and the GFF files can be obtained from NCBI genome database (http://www.ncbi.nlm.nih.gov/genome). The output of the Whole Transcriptome analysis generates (1) a gene counts file, with the base counts summed to a single value across the entire gene length, and with a RPKM value also given for each gene; (2) a BAM file containing the sequence of every mapped read and its mapped location; (3) two pairs of ^*^.wig files (one pair for the two strands on each chromosome) giving the mapped counts at each base position; and (4) a statistics summary on alignment and filtering report. The transcriptomics data are available at the NCBI BioSample database under the accession numbers SAMN02302475–SAMN02302483.

### Metabolites

NMR data was acquired on a Varian Direct Drive (VNMRS) 600 MHz spectrometer (Agilent Technologies) equipped with a Dell Precision T3500 Linux workstation running VNMRJ 3.2. The spectrometer system was outfitted with a Varian triple resonance salt-tolerant cold probe with a cold carbon preamplifier. A Varian standard one dimensional proton nuclear Overhauser effect spectroscopy (NOESY) with presaturation (TNNOESY) was collected on each sample, using the Chenomx standard data collection protocol: non-selective 90° excitation pulse, a 100 ms mixing time, acquisition time of 4 s, spectral width of 12 ppm, and temperature control set to 25°C. A presaturation delay of 1.5 s was used to optimize water suppression. Metabolites analysis was performed using NMR on media as well as cell-free supernatant samples after 60 h of growth. Collected spectra were analyzed using Chenomx 7.6 software (Edmonton, Alberta Canada), with quantifications based on spectral intensities relative to 0.5 mM 2,2-dimethyl-2-silapentane-5-sulfonate, which was added as a spike to each sample.

HPLC was run on a Shimadzu LC-20AD liquid chromatograph with a DGU-20A5 degasser and SIL-20ACHT autosampler, run by a CBM 20A control module. The CTO-20A oven was equipped with an Aminex HPX-87H column and a Biorad Microguard Cation H guard column at 30°C. The machine pumped 0.6 mL/min for the duration of the cycle, with 5 mM H_2_SO_4_ as running buffer. Each injection was 20 μ L and was measured by an RID-10A refractive index detector for 30 min. A xylose standard ran from 0.1% to 1% xylose in water, with an *R*^2^ of 0.9798. The lowest peak was easily visible, and thus our lower detection limit for this study was 0.1%, or 6.25 mM xylose. Samples were run in triplicate.

## Results and discussion

**Figure 1:**
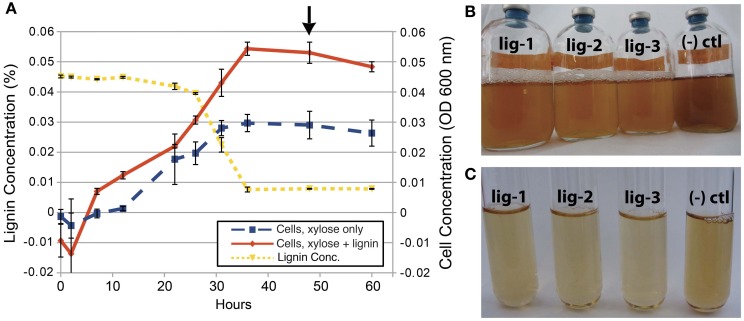
**Anaerobic growth and lignin degradation by *E. lignolyticus* SCF1. (A)** This replicated growth curve experiment (*n* = 3) shows increased cell abundance with lignin, and decreased lignin over time. The arrow denotes the time that samples were collected for transcriptomics, proteomics and metabolomics studies. After 48 h of growth, color change in the lignin media and bubbles indicating CO_2_ gas formation **(B** and **C)** inoculated with SCF1 (bottles lig1–3) is evident when compared to the darker, uninoculated control [“(−) ctl”].

SCF1 is capable of degrading 56% of the lignin under anaerobic conditions within 48 h, with increased cell abundance in lignin-amended compared to unamended growth (Figure 1). Lignin degradation is measured by absorbance at 310 nm, where decreases in absorbance indicate decreasing concentrations of soluble phenolic and polyphenolic compounds (Ahmad et al., 2010). During growth, we also observed color change of the cultures, and production of bubbles that likely signify CO_2_ evolution during the metabolism of the xylose and lignin in the media. We performed experiments to observe lignin degradation during growth on xylose minimal media amended with lignin, because we were unable to detect growth of SCF1 on lignin as sole C source under anaerobic conditions. While this strain was originally isolated growing anaerobically under conditions of minimal agar media with lignin as the sole C source (DeAngelis et al., 2011), the colonies took about 12 weeks to form, and we have been unable to recreate these growth conditions in liquid media for cell biomass sufficient to perform detailed genetic and proteomic analysis. Because of this, genetic, metabolic and proteomic analysis of lignin degradation is performed by comparing lignin-amended xylose minimal media to unamended xylose minimal media, and lignin degradation mechanisms and pathways are inferred by differential gene expression and protein production.

Proteomics analysis produced 7883 unique peptides and 871 unique proteins. Our previous study showed that the SCF1 genome encodes 4449 protein encoding genes (DeAngelis et al., 2011). There were 229 proteins that were significantly differentially abundant between the lignin-amended and unamended growth conditions. Of these, 127 proteins were at least 2-fold up-regulated in the presence of lignin. Pathways with the most hits included proteins associated with metabolism, biosynthesis of secondary metabolites, and ABC transporters (Supplemental Table 2). We further examined proteins and pathways likely associated with xylose degradation, lignin degradation, and dissimilatory lignin reduction to explore the ways in which SCF1 might be gaining a growth advantage in lignin-amended compared to unamended cultivation conditions.

Transcripts were sequenced as 50 bp tags on ABI SOLiD4, and aligned to the SCF1 genome. Data (number of transcripts) was normalized to reads per kilobase of gene per million reads. Of the 4716 genes detected by transcriptomics, 273 were differentially regulated, and 147 were up-regulated in the lignin-amended compared to the xylose only control (Table 1). These included mostly genes associated with metabolism, biosynthesis and transporters (Supplemental Table 3).

**Table 1:** **Proteomic and transcriptomic data and differential regulation in lignin-amended compared to unamended samples**.

	**Unique**	**Significant (*P* < 0.05)**	**Up-regulated**	**Down-regulated**
Peptides	7883	855	626	229
Proteins	869	285	207	79
Transcripts	4716	273	147	126

We chose to analyze both transcripts and proteins after 48 h of anaerobic growth of SCF1 in lignin-amended and unamended xylose minimal media. Sampling during stationary phase was chosen because at this time point, cells had demonstrated lignin degradation, and no further cell growth or significant lignin degradation was observed after around this time. However, we recognize that the choice of stationary phase likely precluded the observation of many transcripts that may have been illuminating for lignin degradation. Indeed, at the gene level, there was little observed overlap between the sequenced transcripts and the observed expressed proteins: of the 871 unique proteins detected, only 11 lignin up-regulated proteins and 4 lignin down-regulated proteins were also observed in the transcripts (Table 2). These constitutively expressed gene products detected by both methods were likely important to growth and survival during the transition into stationary phase, because they had been expressed for lignin degradation and continued to be expressed during transition into stationary phase. For the lignin-amended cultures, the up-regulated and highly transcribed genes included mostly transporters and proteins in the TCA cycle. A carbon starvation protein CstA (Entcl_3779) encoding a predicted membrane protein, also had significantly more transcript and protein in lignin-amended conditions (Schultz and Matin, 1991). The CstA protein is located just upstream of the 4-hydroxyphenylacetate degradation pathway (Entcl_3796-3806), which is also the case for *E. coli* (Prieto et al., 1996). Carbon starvation genes have long been associated with metabolism of aromatic compounds (Blom et al., 1992), and are thought to be a result of membrane toxicity of hydrocarbons that can integrate into cell membranes and cause a leak of the proton motive force (Sikkema et al., 1995). The CstA protein is thought to be involved in transport of nucleic acids, where expression is a hallmark of the cell trying to avoid entry into stationary phase (Schultz and Matin, 1991; Kraxenberger et al., 2012).

**Table 2:** **Genes significantly differentially detected both by transcriptomics and proteomics, where positive fold change in ratios of transcripts or proteins indicates up-regulation in lignin compared to unamended growth, and negative fold-change indicates down-regulation in lignin compared to unamended growth**.

**GeneID**	**Protein description**	**Pathway**	**Fold change for transcripts**	**Fold change for proteins**
Entcl_0332	Phosphoenolpyruvate carboxykinase (ATP) (complement(365954..367573))	Citrate cycle (TCA cycle)	2.670	3.102
Entcl_3179	UspA domain-containing protein (3394773..3395201)	None given	3.080	2.953
Entcl_4175	Periplasmic binding protein/LacI transcriptional regulator (complement(4503494..4504456))	ABC transporters	2.170	2.796
Entcl_3779	Carbon starvation protein CstA (4066791..4068944)	None given	2.670	2.701
Entcl_1304	Malic protein NAD-binding (1376647..1378926)	Pyruvate metabolism	3.770	2.490
Entcl_0617	AI-2 transport system substrate-binding protein (642484..643485)	ABC transporters	3.180	1.780
Entcl_4402	Periplasmic binding protein/LacI transcriptional regulator (complement(4764359..4765249))	ABC transporters	2.020	1.704
Entcl_1207	ABC transporter, substrate-binding protein (complement(1260320..1261303))	ABC transporters	2.380	1.564
Entcl_2658	Isocitrate dehydrogenase, NADP-dependent (complement(2808830..2810080))	Glutathione metabolism	2.010	1.091
Entcl_0176	D-xylose ABC transporter, periplasmic substrate-binding protein (complement(183475..184470))	ABC transporters	2.410	1.035
Entcl_3614	2-oxo-acid dehydrogenase E1 subunit, homodimeric type (complement(3877006..3879669))	Glycolysis/Gluconeogenesis	2.500	−0.229
Entcl_1941	Phosphoribosylglycinamide formyltransferase 2 (complement(2053388..2054566))	Purine metabolism	−2.080	−0.779
Entcl_1559	Cytidine deaminase (complement(1657176..1658060))	Pyrimidine metabolism	−3.710	−1.169
Entcl_0641	Cys/Met metabolism pyridoxal-phosphate-dependent protein (complement(670311..671459))	None given	−2.000	−1.757
Entcl_3443	Taurine dioxygenase (complement(3672816..3673664))	Taurine and hypotaurine metabolism	−14.850	−2.995

Genome sequence analysis of SCF1 had revealed a lack of core protocatechuate and 3-O-methylgallate degradation pathways like those found in *S. paucimobilis* (Masai et al., 2007; Peng et al., 2008). Instead, lignin catabolism seemed likely to proceed via homoprotocatechuate through the 4-hydroxyphenylacetate degradation pathway, a gene cluster that is conserved among the *Enterobacter* and *Klebsiella*. Proteomics supports this, and metabolomics suggests that lignin may also act as a terminal electron acceptor, increasing the growth efficiency on xylose. For these studies, SCF1 was grown in xylose minimal media with and without lignin. All reported differences below have minimum 2-fold changes with significant corrected P-values (Benjamini and Hochberg, 1995).

### Xylose utilization

The SCF1 genome encodes many proteins related to xylose degradation. D-xylose is likely recognized by an ABC related substrate binding protein (SBP) and transported into the cells by ATP-driven ABC transport system. Once inside the cell, xylose isomerase converts it to D-xylose and subsequently converted in to D-xylose 5-phosphate by xylulokinase. D-xylulose 5-phosphate then enters pentose phosphate pathway with the help of certain transketolase enzyme. The proteins D-xylose ABC transporter ATPase and D-xylose ABC transporter periplasmic substrate-binding protein, xylose isomerase, and xylulokinase were all detected in our growth conditions.

**Figure 2:**
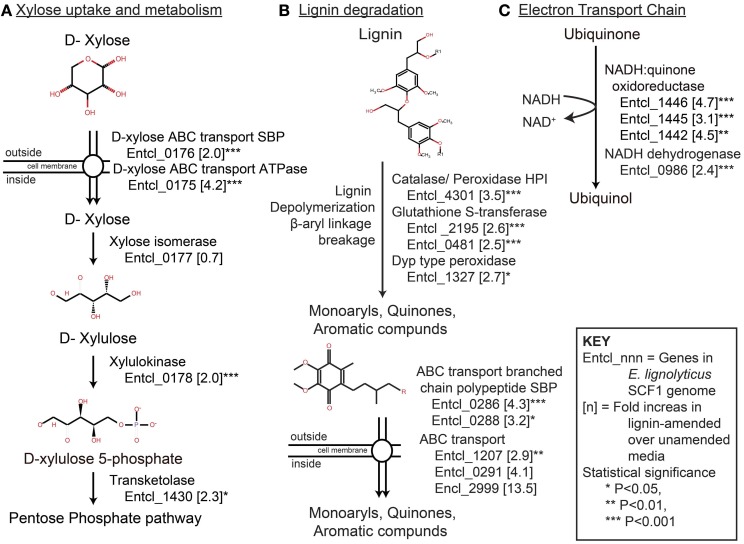
**Pathways associated with (A) xylose degradation, (B) lignin degradation, the 4-hydroxyphenylacetate degradation pathway, a possible pathway of lignin catabolism, and (C) dissimilatory lignin reduction via the electron transport chain.** For each pathway, the number next to the protein ID denotes the fold-level induction in lignin-amended compared to unamended growth conditions. All genes listed were statistically significantly up-regulated in lignin-amended compared to unamended controls; see Table 3 for values.

More efficient xylose utilization in the presence of lignin was suggested by the fact that many proteins associated with xylose uptake and degradation were significantly up-regulated in the lignin-amended compared to the unamended controls (Table 3, Figure 2A). Xylose transport system proteins were significantly up-regulated, as were both ATPase transport and SBPs related to D-xylose ABC type transport system: D-xylose ABC transporter ATPase subunit (Entcl_0175) and D-xylose ABC transporter periplasmic SBP (Entcl_0176). While the expression of xylose isomerase (Entcl_0177) was detected but not significantly up-regulated in our lignin-amended sample, xylulokinase (Entcl_0178) was significantly up-regulated in the lignin treated sample. Various proteins related to transketolase were also up-regulated in lignin-amended sample (Entcl _0820, Entcl_1430, and Entcl_1431), though only transketolase (Entcl_1430) was significant. Adav et al. (2012) has shown up-regulation of xylose isomerase in the secretome of the thermostable filamentous bacteria *Thermobifida fusca* when grown on different lignocellulosic biomass. As our proteomics were performed on cell pellets, it is possible that secretomes were either missed or not induced due to the soluble nature of lignin. Adav et al. also showed expression of different ABC type-sugar transport systems depended upon the type of lignocellulosic biomass *T. fusca* was grown on, consistent with our observations of up-regulated ABC transporters.

**Table 3:** **Proteins over-expressed in lignin-amended compared to unamended controls**.

**Locus Tag**	**Protein Description**	**Pathway**	**Fold change**	***p*-value**
**XYLOSE DEGRADATION**				
Entcl_0175	D-xylose ABC transporter ATPase subunit	ABC transporters	4.2	2.5e-08
Entcl_0176	D-xylose ABC transporter periplasmic SBP	ABC transporters SBP	2.0	2.1e-10
Entcl_0178	Xylulokinase	Xylose degradation I	2.0	2.0e-04
Entcl_1430	Transketolase	Pentose phosphate	2.3	4.2e-02
Entcl_0081	Glycoside hydrolase family 31	–	2.6	7.4e-10
**PUTATIVE LIGNIN DEGRADATION**				
**Peroxidase**				
Entcl_4301	Catalase/Peroxidase HPI	Tryptophan metabolism	3.5	1.5e-29
Entcl_1327	Dyp-type peroxidase family	–	2.7	1.5e-02
**β-aryl linkage**				
Entcl_2195	Glutathione S-transferase domain	Glutathione metabolism	2.6	4.3e-12
Entcl_0481	Glutathione S-transferase domain	Glutathione metabolism	2.5	9.2e-04
**LIGNIN AS ELECTRON ACCEPTOR**				
Entcl_1442	NADH:quinone oxidoreductase B subunit	Electron transport	4.5	4.2e-03
Entcl_1445	NADH:quinone oxidoreductase F subunit	Electron transport	3.1	1.8e-04
Entcl_1446	NADH:quinone oxidoreductase G subunit	Electron transport	4.7	3.6e-22
Entcl_0986	NADH dehydrogenase (ubiquinone)	Electron transport	2.4	2.3e-04
Entcl_0361	Nitrite reductase [NAD(P)H)]	Electron transport	3.5	1.8e-04
Entcl_2895	DMSO reductase subunit A	Electron transport	2.7	3.0e-12
**Transporters**				
Entcl_4417	ATP synthase F0, β subunit	Energy metabolism	2.5	3.4e-04
Entcl_4419	ATP synthase F1, α subunit	Energy metabolism	2.2	4.8e-12
Entcl_0286	Branched chain polypeptide extracellular SBP	ABC transport SBP	4.3	6.2e-20
Entcl_0288	Branched chain polypeptide extracellular SBP	ABC transport SBP	3.2	1.9e-02
Entcl_1207	ABC transporter	ABC transport	2.9	1.0e-03

All listed were either 2-fold over-expressed or greater (Ratio) or had a significant p-value.

Because we observed reproducible increased cell abundance on xylose minimal media amended with lignin compared to controls, we also looked for evidence of increased efficiency in respiration, hypothesizing that SCF1 may be using lignin as a terminal electron acceptor and thus increasing its efficiency of growth. After 60 h of growth, we observed no difference in xylose remaining in the media by NMR, but we detected significantly higher levels of acetate and formate produced in the lignin amended media compared to the unamended control (Table 4). However, differences in metabolites in lignin-amended media (no cells) compared to unamended revealed that the lignin may obscure some of the NMR signals of metabolites, so we analyzed xylose concentrations using HPLC. HPCL is not as sensitive (detection limits are in the mM range, compared to NMR which has limits in the μ M range), but there is no interference of lignin. HPLC demonstrated that both lignin-amended and unamended samples were degrading xylose. After 48 h the lignin-amended samples had 5% less measurable xylose compared to the unamended samples (0.703 ± 0.012% xylose in the xylose only growth conditions, compared to 0.667 ± 0.012% xylose in the lignin-amended growth conditions, *P* = 0.09). This could suggest that the degradation of lignin somehow aids in the breakdown of xylose, which may support lignin as a terminal electron acceptor.

**Table 4:** **Metabolite analysis based on NMR of supernatants for SCF1 grown in xylose minimal media with and without lignin**.

	**Xylose only media**	**Xylose + lignin media**	***P***	**Cells + Xylose only**	**Cells + Xylose + lignin**	***P***
Xylose	47352 ± 1380	51464 ± 541	^**^	59512 ± 4948	67402 ± 1068	n.s.
Acetate	22.0 ± 3	3.0 ± 0.1	^**^	841 ± 51.2	1340 ± 126	^*^
Ethanol	175 ± 32	122 ± 30	^**^	6715 ± 4699	4788 ± 624	n.s.
Formate	161 ± 2.6	110 ± 4.7	^**^	1625 ± 149	1908 ± 0	^***^

Averages are listed (n = 3), and P-values are denoted as not significant (n.s.s),

* P < 0.05,

** P < 0.01,

*** P < 0.001. All concentrations are in μ M.

### Lignin degradation

Because lignin concentrations based on absorbance decreased significantly over the course of SCF1 growth, we expected to find lignin degradation pathway proteins up-regulated in the lignin-amended compared to the unamended controls. We identified SCF1 homolog targets that have been implicated in other lignin or poly-phenolic degrading bacteria. Targets consisted of enzymes associated with lignin or polyphenolic degradation, and other genes that might be involved in sugar utilization (Ramachandra et al., 1988; Harwood and Parales, 1996; Masai et al., 2007; Rakotoarivonina et al., 2011). This included the enzymes of the protocatechuate pathway found in *S. paucimobilis* (Masai et al., 2007), proteins of the protocatechuate pathway conserved among *Pseudomonas*, *Acinetobacter*, and *Arthrobacter* species (Harwood and Parales, 1996), a *Thermobacillus xylanilyticus* feruloyl esterase and two hypothetical β-aryl esterases from *Bacillus clausii* (Rakotoarivonina et al., 2011), and extracellular lignin peroxidase from *Streptomyces viridosporus* (Ramachandra et al., 1988). A commonly found bond in the complex heteropolymer lignin is the diphenyl, a simplified type of di-aryl ether bond, which should be degraded by phenol oxidase, peroxidase or laccase enzymes (Ramachandra et al., 1988; Chang, 2008). Based on our initial genomics analysis and reports of other lignin-degrading microbes, we identified the 4-hydroxyphenylacetate degradation pathway, catalase/peroxidase enzymes, and the glutathione biosynthesis and GST pathways as likely implicated in SCF1 lignin degradation.

The catabolite 4-hydroxyphenylacetate is an intermediate in the degradation of lignin monomers (Grbić-Galić, 1985), and can be degraded under anaerobic conditions by a number of denitrifying and sulfate-reducing bacteria (Heider and Fuchs, 1997; Gibson and Harwood, 2002). In this pathway, 4-hydroxyphenylacetate is degraded into the TCA cycle intermediate succinate and in this way provides energy to the bacteria (Martín et al., 1991). The SCF1 genome encodes the entire 4-hydroxyphenylacetate degradation pathway gene in a single gene cluster HpaRGEDFHIXABC (DeAngelis et al., 2011). Protein abundance data showed several proteins typically associated with this pathway activated under lignin-amended samples. Proteins encoded by HpaE (Entcl_3798) and HpaG (Entcl_3797) genes were present in lignin-amended sample.

Lignin degradation has been extensively studied in fungi, which produce extracellular peroxidases/catalase that are able to degrade lignin (Wong, 2009). Similarly, several published studies also report soil bacteria that are able to degrade lignin with the use of catalase or peroxidase enzymes. *Streptomyces viridosporous*, *Nocardia autotrophica*, and *Rhodococcus* sp. are well studied aerobic lignin degrading bacteria that produce extracellular peroxidase (Zimmermann, 1990). We found two peroxidase type proteins which are significantly up-regulated in lignin-amended sample: catalase/peroxidase HPI (Entcl_4301) and DypB-type peroxidase (Entcl_1327) (Figure 2B). The dyp type peroxidase protein family was identified in *Rhodococcus jostii* RHA1 (Ahmad et al., 2011) and was suggested for lignin degradation by β-aryl ether breakdown. This enzyme is activated by Mn^2+^ ions and was shown to degrade lignin and produce monoaryl like 2, 6-dimethaoxybenzoquinone (Singh et al., 2013). However, the nature of the involvement of peroxide in anaerobic lignin degradation is still unclear.

We expected to find strong phenol oxidase and peroxidase activity in SCF1, because it was isolated from the Luquillo LTER soils, where soil phenol oxidase and peroxidase activities were detected across an elevational gradient spanning 2.5 km (Silver et al., 1999, in press). Soils from the Short Cloud Forest site (SCF) were highest in phenol oxidase and peroxidase activity compared to the lower elevation, fluctuating redox and aerobic sites (DeAngelis et al., 2013). Though L-DOPA is an inexpensive and easily detectable assay for cell cultures, it has been criticized as a poor soil assay substrate because it is susceptible to chemical oxidation (Sinsabaugh, 2010), which likely comprised some of the background activity we detected in our soils (DeAngelis et al., 2013). Enzyme activity analysis of SCF1 using L-DOPA as a substrate revealed no peroxidase production, or phenol oxidase production, under aerobic and anaerobic conditions. We also used ABTS as a substrate and detected phenol oxidase activity at 3.3 mU (10^6^ cells)^−1^, and peroxidase activity at 2.3 mU (10^6^ cells)^−1^. These rates potentially support a pathway for lignin degradation that includes catalase and peroxidase enzymes, but further study will be required to understand if these proteins are expressed anaerobically as well as aerobically. However, the enzyme assay method will continue to be hindered by substrate specificity, where there are many substrates in nature and available for analysis (Mayer and Staples, 2002; Sinsabaugh, 2010).

GST has been studied as a method of detoxification metabolism in eukaryotes (Yin et al., 2000; Cho et al., 2001). A few Proteobacteria genomes also contain large sets of GST genes and are known to be involved in the degradation of aromatic compounds (Lloyd-Jones and Lau, 1997; Vuilleumier and Pagni, 2002). GST has been shown to have etherase activity and involved in β-aryl ether cleavage in lignin degradation in *S. paucimobilis* SYK-6 (Masai et al., 1999, 2007). The activity of GST for lignin degradation is enhanced by the addition and presence of glutathione (Masai et al., 1993). Glutathione synthesis from its precursor glutamate takes place in the cytosol, and we found glutamate/cysteine ligase (Entcl_1035) and glutathione synthetase (Entcl_0809) proteins involved in glutathione biosynthesis expressed in our cultures, though with no difference in abundance between lignin-amended and unamended growth conditions (Figure 2B). We also found ABC transport related to glutamate/aspartate transport system (Entcl_3149) up-regulated in lignin-amended samples. Similarly, different sets of GST protein (Entcl_2195 and Entcl_0481) and ABC transport related glutathione transport system (Entcl_2986) were significantly up-regulated in lignin-amended sample. Thus, the presence of glutathione biosynthesis proteins and transport system, and GST protein and its transport system could suggest a possible mechanism of lignin depolymerization by β-aryl ether cleavage in lignin-amended sample.

### Dissimilatory lignin reduction

It is possible that SCF1 is using lignin as a terminal electron acceptor, and in this way degrading lignin in a dissimilatory manner. Various substituted quinones have been identified as key intermediates in the degradation of lignin model compounds (Ander et al., 1980; Buswell and Eriksson, 1988; Schmidt et al., 1989). These intermediates include substituted quinones, hydroquinones, benzaldehydes, benzoic acids, and ring-opened fragments (Buswell and Eriksson, 1988; Higuchi et al., 1990). Because lignin is a complex heteropolymeric molecule, it is possible that any of these intermediates could exist as analogous moieties and be used by the SCF1 as a terminal electron acceptor. Intracellular NADH-quinone oxidoreductase reduces 2-methoxyquinone and several other substituted quinones to their hydroquinones (Buswell et al., 1979; Buswell and Eriksson, 1988). Quinones have been studied as potential electron acceptor in anaerobic environment by facultative anaerobes (Newman and Kolter, 2000) and are important electron-accepting groups in humic substances (Scott et al., 1998). While lignin is made up of only three monolignol builfinh blocks, including coniferyl alcohol, sinapyl alcohol, and p-coumaryl alcohol, they are polymerized during biosynthesis in the plant by way of oxidative radicalization and coupling of phenols, which creates a wide variety of molecular moieties available for reduction or depolymerization via biotic degradation (Vanholme et al., 2010). Because of this variety, NMR analysis would be required to both elucidate the structure of the lignin as well as the chemical characters of the reduced and possibly depolymerization products that result from SCF1 degradation. We have applied proteomics to elucidate the reduction pathways of SCF1 in lignin-amended vs. unamended growth on xylose minimal media.

We found three NADH-quinone oxidoreductase proteins (Entcl_1446, Entcl_1442, and Entcl_1445) significantly up-regulated in lignin amended samples (Figure 2C). These proteins are integral in electron transport chain (Brandt, 2006) and are involved in transfer of electron from NADH to quinone like molecule as electron acceptor. Since lignin may be a precursor to humic substances, we assume degradation of lignin may result in quinone molecules used as electron acceptors to harvest the energy for microbial respiration. These reduced seimiquinones abiotically transfer electrons between dehydrogenase and the reductase enzyme, and this electron transfer would yield energy for bacterial growth (Scott et al., 1998). We also found significant up-regulation of NADH dehydrogenase (Entcl_0986), nitrite reductase (Entcl_0361) and DMSO reductase (Entcl_2895) in lignin amended sample. NADH serves as the electron donor, nitrite/DMSO as the electron acceptor and seimiquinones as mediator and could form a modular electron transport chain.

We assume the addition of lignin is enhancing efficiency of energy production in SCF1 in lignin-amended samples. This was distinct from high cell abundance and high growth of SCF1 in treatment samples. Addition of vanillin, an intermediate during fungal lignin degradation, has shown to enhance energy productions in basidomycetes which seem to be required for xenobiotic metabolism and as well for cell growth (Shimizu et al., 2005). Enhanced energy production in this study was related to the up-regulation of ATP synthase. We also found proteins related to various subunits of ATP synthase F0/F1 (Entcl_4417, Entcl_4418, Entcl_4419, Entcl_4420, and Entcl_4421). Significant up-regulation of ATP synthase in lignin-amended sample could be justified as SCF1 may require more energy to overcome the high energy barrier for ring reduction in lignin.

The transport of small aromatic molecules after lignin degradation is important because these small molecules likely account for a significant source of energy and biomass among lignin-degrading microbes (Michalska et al., 2012). Aromatic compounds derived from lignin degradation could be imported by an ATP-depended mechanism (Paulsen et al., 2000; Chaudhry et al., 2007). These transportations are mediated by ATP-binding cassette (ABC) transporters. The bacterial ABC transporter is composed of a transmembrane permease, a cytoplasmic ATPase subunit, and a periplasmic solute-binding protein (SBP) (Michalska et al., 2012). In known lignin degrading bacteria, these SBPs are identified as branched-chain amino acid-binding proteins (Giuliani et al., 2008; Oda et al., 2008). In *Rhodopseudomonas palustris*, a cluster of ABC transporter genes are likely involved in the uptake of benzoate into cells (Egland et al., 1997). This bacterium also contains several periplasmic binding-protein components of an ABC system involved in active transport for lignin-derived aromatic substrates (Salmon et al., 2013). We have also found significant up-regulation of an ABC transporter (Entcl_1207) and branched chain polypeptide extracellular ligand-binding receptor (Entcl_0286 and Entcl_0288) in lignin amended samples. These ABC system proteins with SBP could be involved in active transportation of lignin derived simpler aromatic compounds into the cells after degradation by putative lignin degrading proteins produced by SCF1.

While the proteomics and metabolomics data support the hypothesis that lignin is being used by the SCF1 as an additional terminal electron acceptor as well as a C source, we wanted to rule out the possibility that were contaminants in the lignin that might contribute to the observed increased cell growth and activity. By HPLC, no sugar peaks or peaks of any size appeared after 7.5 min, specifically none between 9 and 13 min, where any sugars should appear. For example, glucose runs at 10.16 min, fructose at 10.39, xylose at 10.39, rhamnose at 11.20, and arabinose at 11.34 min. The detection limit of the HPLC is in the mM range for sugars. We also used NMR to test the media for sugars. Only xylose was detected, and although there was significantly more xylose detected in the lignin-amended compared to the unamended samples (51.7 ± 2.95 mM xylose in the lignin-amended media, 47.4 ± 5.4 mM unamended xylose minimal media, mean ± standard deviation, *P* < 2e-5), NMR did not detect any other sugars, with detection limits in the μ M range. NMR may also be subject to peak interference of lignin, suggesting that increased xylose detection is an artifact. Metabolomics analysis of the media by HPLC and NMR both showed that it is extremely unlikely that the increased cell biomass and microbial activity were due to sugar contamination in the lignin. In addition, the increased production of proteins in the hydroxyphenylacetate pathway, analogous to pathways of lignin degradation observed for other bacteria, further support the hypothesis that SCF1 is using lignin in both assimilatory and dissimilatory pathways.

Despite the molecular microbial evidence that *E. lignolyticus* SCF1 is able to use lignin in both assimilatory and dissimilatory pathways, there are still unanswered questions. For one, the products of SCF1 anaerobic lignin reduction remain unclear. These products could include phenolic aldehyde, acid, or ketone monomers that are observed to be released during alkaline CuO oxidation (Thevenot et al., 2010), or any of the catabolic pathway intermediates that have observed during anaerobic lignin degradation of other bacteria, such as the catabolic pathways described for degradation of lignin and lignin-derived compounds in *S. paucimobilis* SYK-6 (Masai et al., 2007) and others (Harwood and Parales, 1996; DeRito et al., 2005; McLeod et al., 2006; Bugg et al., 2011b; Huang et al., 2013). The use of lignin dimers or model lignin compounds such as artificial or naturally occurring aromatics would permit measurement of specific rates of degradation of specific bonds present in lignin (Kato et al., 1998; Koga et al., 1999; Chang, 2008). However, dissimilatory reduction of the complex heteropolymer lignin might result in increased saturation of bonds or hydrolysis of end groups, which would not result in production small molecules. To make these measurements would require high resolution molecular analysis using NMR, mass spectrometry or FTIC, where specific structural details of chemical bonds and end groups indicative of specific breakdown products can be identified (Morreel et al., 2010; Vanholme et al., 2010). These methods in combination with tracer experiments using ^13^C labeled lignin should be used in the future to determine specific degradation pathways and moieties of lignin that are released. For example, growth of *Fibrobacter succinogenes* S85 on ^13^C-wheat straw revealed succession of different fractions of wheat straw without preferential degradation of amorphous vs. crystalline cellulose (Matulova et al., 2005). This type of study would strongly advance our understanding of anaerobic bacterial lignin degradation, though currently ^13^C-lignin studies seem to be concentrated on determining the structure of lignin, which may preclude knowing degradation products in detail (Morreel et al., 2010; Foston et al., 2012). Finally, the investigation of a single time point potentially masked detection of other degradation pathways or control points that would have been evident in early or mid logarithmic growth, before significant lignin had been degraded. An examination of the transcripts and proteins over a time-course of lignin degradation should be analyzed in order to link the controls over initiation and termination of assimilatory and dissimilatory lignin degradation.

## Conclusions

Previous work has shown that *E. lignolyticus* SCF1 possesses a suite of membrane pumps that confer tolerance to high concentrations of both salt and ionic liquids, which are used as an alternative pre-treatment for lignin removal in plant feedstock material (Khudyakov et al., 2012). We also know that SCF1 is derived from a wet tropical forest soil environment that is characterized by low and fluctuating redox conditions as well as very fast rates of litter decomposition (Parton et al., 2007; Silver et al., in press). This work shows that *E. lignolyticus* SCF1 is able to use lignin in both assimilatory and dissimilatory pathways, where assimilatory pathways are glycolysis and the pentose phosphate pathway, and dissimilatory reduction seem to occur by oxidative phosphorylation via the electron transport chain. Dissimilatory reduction of lignin-model compounds and aromatics has been well established (Harwood and Parales, 1996), as has the ability for a range of bacteria to shuttle electrons via quinones and soluble humic substances (Newman and Kolter, 2000). It is also remarkable that SCF1 is able to grow so well in the presence of lignin, which contains many soluble products that have proven to be inhibitory to growth of many other organisms including popular model organisms for metabolic engineering such as *E. coli*. While there are many studies that demonstrate degradation of lignin for assimilatory pathways (Bugg et al., 2011a), this is the first to demonstrate both assimilatory and dissimilatory reduction of the complex heteropolymer plant lignin by a soil bacterium.

### Conflict of interest statement

The authors declare that the research was conducted in the absence of any commercial or financial relationships that could be construed as a potential conflict of interest.

## Supplementary material

Supplementary Table 1 **Changes in mobile phase for each 2D-LC fraction**.

Supplementary Table 2 **Summary of proteins annotated to metabolic pathways, the average and standard deviation of the effect of lignin on the proteins in each pathway, and the number of proteins total in each pathway**.

Supplementary Table 3 **Summary of transcripts annotated to metabolic pathways and the number of transcripts total in each pathway**.
