# Supplementary material for: Evidence supporting dissimilatory and assimilatory lignin degradation in Enterobacter lignolyticus SCF1
Source: Front Microbiol. 2013 Sep 19;4:280. doi: 10.3389/fmicb.2013.00280 (PMC3777014; doi:10.3389/fmicb.2013.00280)
Supplement: Supplementary Table 1 — Changes in mobile phase for each 2D-LC fraction. [file DataSheet1.DOC]

**Supplemental Table 1**. Changes in mobile phase for each 2D-LC fraction.

| SCX Fractionation 1st dimension | | |  | Reversed-phase separation 2nd dimension | | |
| --- | --- | --- | --- | --- | --- | --- |
| Fraction | %B Start | %B End |  | Time (min) | %B | Event |
| 1 | 0 | 0 |  | -40 | 0 | Trap/Wash |
| 2 | 0 | 0.5 |  | 0 | 0 |  |
| 3 | 0.5 | 1.5 |  | 2 | 8 |  |
| 4 | 1.5 | 3 |  | 10 | NA | Start Acq. |
| 5 | 3 | 5 |  | 20 | 12 |  |
| 6 | 5 | 7 |  | 75 | 35 |  |
| 7 | 7 | 10 |  | 97 | 60 |  |
| 8 | 10 | 13 |  | 100 | 85 |  |
| 9 | 13 | 19 |  | 103 | 0 |  |
| 10 | 19 | 25 |  | 110 | NA | End Acq. |
| 11 | 25 | 32 |  |  |  |  |
| 12 | 32 | 39 |  |  |  |  |
| 13 | 39 | 42 |  |  |  |  |
| 14 | 42 | 50 |  |  |  |  |
| 15 | 50 | 99 |  |  |  |  |
