# Supplementary material for: Evidence supporting dissimilatory and assimilatory lignin degradation in Enterobacter lignolyticus SCF1
Source: Front Microbiol. 2013 Sep 19;4:280. doi: 10.3389/fmicb.2013.00280 (PMC3777014; doi:10.3389/fmicb.2013.00280)
Supplement: Supplementary Table 2 — Summary of proteins annotated to metabolic pathways, the average and standard deviation of the effect of lignin on the proteins in each pathway, and the number of proteins total in each pathway. [file DataSheet2.DOC]

**Supplemental Table 2**. Summary of proteins annotated to metabolic pathways, the average and standard deviation of the effect of lignin on the proteins in each pathway, and the number of proteins total in each pathway.

| **Pathway** | **Avg lignin effect** | **Std dev lignin effect** | **Sum of proteins per pathway** |
| --- | --- | --- | --- |
| none given | 0.679 | 2.161 | 36 |
| Microbial metabolism in diverse environments | 1.273 | 5.760 | 11 |
| ABC transporters | 1.426 | 5.628 | 6 |
| Glyoxylate and dicarboxylate metabolism | 1.667 | 6.708 | 6 |
| Arginine and proline metabolism | -1.704 | 6.793 | 3 |
| Cysteine and methionine metabolism | -0.704 | 7.350 | 3 |
| Galactose metabolism | -0.889 | 3.008 | 3 |
| Methane metabolism | 3.407 | 7.185 | 3 |
| Selenocompound metabolism | -0.259 | 6.971 | 3 |
| Two-component system | -6.444 | 6.087 | 3 |
| Alanine, aspartate and glutamate metabolism | -1.444 | 9.585 | 2 |
| Amino sugar and nucleotide sugar metabolism | -2.556 | 2.514 | 2 |
| Citrate cycle (TCA cycle) | 5.944 | 5.893 | 2 |
| Glutathione metabolism | -1.889 | 0.471 | 2 |
| Glycerolipid metabolism | -7.444 | 3.300 | 2 |
| Lipopolysaccharide biosynthesis | 0.722 | 0.079 | 2 |
| Pentose and glucuronate interconversions | -3.833 | 2.278 | 2 |
| Bacterial chemotaxis | 4.333 | n.a. | 1 |
| DNA replication | 1.222 | n.a. | 1 |
| Flagellar assembly | -11.444 | n.a. | 1 |
| Glycine, serine and threonine metabolism | -3.111 | n.a. | 1 |
| Glycolysis / Gluconeogenesis | 10.111 | n.a. | 1 |
| Homologous recombination | 1.222 | n.a. | 1 |
| Lysine degradation | 1.778 | n.a. | 1 |
| Mismatch repair | 1.222 | n.a. | 1 |
| Nitrogen metabolism | -8.222 | n.a. | 1 |
| Nitrotoluene degradation | -5.111 | n.a. | 1 |
| One carbon pool by folate | -4.667 | n.a. | 1 |
| Pentose phosphate pathway | -2.222 | n.a. | 1 |
| Phenylalanine metabolism | -2.667 | n.a. | 1 |
| Phenylalanine, tyrosine and tryptophan biosynthesis | 1.000 | n.a. | 1 |
| Propanoate metabolism | 6.333 | n.a. | 1 |
| Pyruvate metabolism | 10.111 | n.a. | 1 |
| Ribosome | 0.333 | n.a. | 1 |
| Sulfur metabolism | -5.222 | n.a. | 1 |
| Taurine and hypotaurine metabolism | -3.778 | n.a. | 1 |
| Thiamine metabolism | -3.889 | n.a. | 1 |
| Valine, leucine and isoleucine degradation | 6.333 | n.a. | 1 |
