# Supplementary material for: Evidence supporting dissimilatory and assimilatory lignin degradation in Enterobacter lignolyticus SCF1
Source: Front Microbiol. 2013 Sep 19;4:280. doi: 10.3389/fmicb.2013.00280 (PMC3777014; doi:10.3389/fmicb.2013.00280)
Supplement: Supplementary Table 3 — Summary of transcripts annotated to metabolic pathways and the number of transcripts total in each pathway. [file DataSheet3.DOC]

**Supplemental Table 3.** Summary of transcripts annotated to metabolic pathways and the number of transcripts total in each pathway.

| **Pathway Short Name** | **Number Of Transcripts** |
| --- | --- |
| Metabolic pathways | 403 |
| Biosynthesis of secondary metabolites | 168 |
| Microbial metabolism in diverse environments | 140 |
| ABC transporters | 138 |
| Two-component system | 101 |
| Biosynthesis of amino acids | 72 |
| Aminoacyl-tRNA biosynthesis | 58 |
| Ribosome | 49 |
| Purine metabolism | 47 |
| Nitrogen metabolism | 40 |
| Phosphotransferase system (PTS) | 37 |
| Flagellar assembly | 36 |
| Glycolysis / Gluconeogenesis | 32 |
| Pyrimidine metabolism | 32 |
| Pyruvate metabolism | 30 |
| Porphyrin and chlorophyll metabolism | 30 |
| Arginine and proline metabolism | 29 |
| Amino sugar and nucleotide sugar metabolism | 29 |
| Fructose and mannose metabolism | 29 |
| Bacterial chemotaxis | 26 |
| Propanoate metabolism | 25 |
| Starch and sucrose metabolism | 24 |
| Pentose and glucuronate interconversions | 24 |
| Glycine, serine and threonine metabolism | 23 |
| Pentose phosphate pathway | 22 |
| Methane metabolism | 20 |
| Cysteine and methionine metabolism | 19 |
| Glycerophospholipid metabolism | 19 |
| Butanoate metabolism | 18 |
| Oxidative phosphorylation | 18 |
| Alanine, aspartate and glutamate metabolism | 18 |
| Glyoxylate and dicarboxylate metabolism | 17 |
| Galactose metabolism | 16 |
| Phenylalanine metabolism | 16 |
| Phenylalanine, tyrosine and tryptophan biosynthesis | 15 |
| Glutathione metabolism | 15 |
| Homologous recombination | 14 |
| Selenocompound metabolism | 14 |
| Sulfur metabolism | 13 |
| Mismatch repair | 13 |
| Base excision repair | 12 |
| Glycerolipid metabolism | 12 |
| Fatty acid biosynthesis | 12 |
| Citrate cycle (TCA cycle) | 12 |
| Lipopolysaccharide biosynthesis | 12 |
| Valine, leucine and isoleucine degradation | 11 |
| Fatty acid metabolism | 11 |
| Histidine metabolism | 11 |
| Folate biosynthesis | 11 |
| Peptidoglycan biosynthesis | 10 |
| Biotin metabolism | 10 |
| Sulfur relay system | 9 |
| One carbon pool by folate | 9 |
| Ascorbate and aldarate metabolism | 9 |
| beta-Alanine metabolism | 9 |
| Ubiquinone and other terpenoid | 9 |
| 2-Oxocarboxylic acid metabolism | 9 |
| Tryptophan metabolism | 8 |
| Benzoate degradation | 8 |
| Inositol phosphate metabolism | 8 |
| Nicotinate and nicotinamide metabolism | 8 |
| Pantothenate and CoA biosynthesis | 7 |
| Terpenoid backbone biosynthesis | 7 |
| Lysine degradation | 7 |
| Streptomycin biosynthesis | 7 |
| Vitamin B6 metabolism | 6 |
| Protein export | 6 |
| RNA degradation | 6 |
| Lysine biosynthesis | 6 |
| Tyrosine metabolism | 6 |
| Bacterial secretion system | 6 |
| DNA replication | 6 |
| Valine, leucine and isoleucine biosynthesis | 6 |
| Cyanoamino acid metabolism | 5 |
| Nucleotide excision repair | 5 |
| Thiamine metabolism | 5 |
| Aminobenzoate degradation | 5 |
| Taurine and hypotaurine metabolism | 5 |
| Limonene and pinene degradation | 4 |
| Synthesis and degradation of ketone bodies | 4 |
| Nitrotoluene degradation | 4 |
| Chloroalkane and chloroalkene degradation | 4 |
| Biosynthesis of unsaturated fatty acids | 4 |
| Riboflavin metabolism | 4 |
| C5-Branched dibasic acid metabolism | 4 |
| Polyketide sugar unit biosynthesis | 4 |
| Phosphonate and phosphinate metabolism | 4 |
| Arachidonic acid metabolism | 3 |
| D-Glutamine and D-glutamate metabolism | 3 |
| D-Alanine metabolism | 3 |
| Xylene degradation | 3 |
| Other glycan degradation | 3 |
| Caprolactam degradation | 3 |
| Geraniol degradation | 3 |
| Dioxin degradation | 3 |
| RNA polymerase | 2 |
| Novobiocin biosynthesis | 2 |
| Naphthalene degradation | 2 |
| Biosynthesis of siderophore group nonribosomal peptides | 1 |
| Sphingolipid metabolism | 1 |
| alpha-Linolenic acid metabolism | 1 |
| Toluene degradation | 1 |
